# The 2019 US medical genetics workforce: a focus on clinical genetics

**DOI:** 10.1038/s41436-021-01162-5

**Published:** 2021-05-03

**Authors:** Brittany D. Jenkins, Catherine G. Fischer, Curt A. Polito, Deborah R. Maiese, Alisha S. Keehn, Megan Lyon, Mathew J. Edick, Matthew R. G. Taylor, Hans C. Andersson, Joann N. Bodurtha, Miriam G. Blitzer, Maximilian Muenke, Michael S. Watson

**Affiliations:** 1 Cancer Prevention Fellowship Program, Division of Cancer Prevention, National Cancer Institute, Bethesda, MD USA; 2 McKusick-Nathans Dept. of Genetic Medicine, Johns Hopkins University, Baltimore, MD USA; 3 American College of Medical Genetics and Genomics, Bethesda, MD USA; 4 American College of Medical Genetics and Genomics; Health Resources and Services Administration/Maternal Child Health Bureau (as of 11/8/2020), Rockville, MD USA; 5 Michigan Public Health Institute, Okemos, MI USA; 6 University of Colorado School of Medicine, Aurora, CO USA; 7 Tulane University Medical Center, New Orleans, LA USA; 8 American Board of Medical Genetics and Genomics, Rockville, MD USA

## Abstract

**Purpose:**

This study characterizes the US clinical genetics workforce to inform workforce planning and public policy development.

**Methods:**

A 32-question survey was electronically distributed to American Board of Medical Genetics and Genomics board-certified/eligible diplomates in 2019. We conducted a descriptive analysis of responses from practicing clinical geneticists.

**Results:**

Of the 491 clinical geneticists responding to the survey, a majority were female (59%) and White (79%), worked in academic medical centers (73%), and many engaged in telemedicine (33%). Clinical geneticists reported an average of 13 new and 10 follow-up patient visits per week. The average work week was 50 hours and the majority (58%) worked over half-time in clinical duties. Providers indicated that 39% of new emergency patients wait 3 days or more, and 39% of nonemergency patients wait over 3 months to be seen. Respondents were geographically concentrated in metropolitan areas and many reported unfilled clinical geneticist job vacancies at their institution of more than 3 years.

**Conclusion:**

With the rapid expansion of genomic medicine in the past decade, there is still a gap between genetics services needed and workforce capacity. A concerted effort is required to increase the number of clinical geneticists and enhance interdisciplinary teamwork to meet increasing patient needs.

## INTRODUCTION

Genomic medicine applies our scientific understanding of genes and the environment to diagnose, treat, manage, and prevent disease in individuals and families.^1^ The medical genetics workforce includes clinical and laboratory geneticists, genetic counselors, genetic nurses, metabolic dietitians, and other genetic health-care professionals. These providers serve in a variety of practice settings including clinical, research, education, and health-care management. Members of the genetics workforce provide a comprehensive range of services including genetic and genomic testing; diagnosis, treatment, and prevention of genetic disease; counseling for patients and their families; and consultations with other medical professionals. Other health professionals not specifically trained in medical genetics may independently interact with patients who have or are at risk of genetic disease, but their scope of practice related to genetics services is less well-defined and their comfort with delivering these services varies.^2–5^

Medical geneticists have advanced training (MD, DO, PhD, or equivalent) and receive specialty certification through the American Board of Medical Genetics and Genomics (ABMGG). Currently, ABMGG offers three specialty certifications: Clinical Genetics and Genomics (CGG), Clinical (Laboratory) Biochemical Genetics (CBG), and Laboratory Genetics and Genomics, a new specialty that is a merger of the specialties of Clinical Cytogenetics and Genomics (CCyG) and Molecular Genetics and Genomics (MGG). In addition, subspecialty certification for MDs is offered in Medical Biochemical Genetics (MBG) and in Molecular Genetic Pathology, the latter of which is jointly offered by the ABMGG and the American Board of Pathology. The important roles of the other members of the interdisciplinary team seeing genetics patients and their workforce issues have been previously addressed.^6,7^

The last major medical genetics workforce survey was conducted in 2003 at the completion of the Human Genome Project.^8,9^ This survey of all ABMGG-certified medical geneticists plus a series of Banbury conferences^10–12^ revealed a critical shortage of qualified genetics health-care professionals that created barriers to care for two main reasons. First, the workforce itself was shrinking—medical geneticists were exiting largely due to retirement, and an insufficient number of newly trained medical geneticists were entering the workforce to achieve growth. Second, the need for medical geneticists had been increasing due to technological advances and expanded clinical applications. These trends resulted in significant challenges in patient access to genetic services in many areas of the United States.

Since these reports were published more than 15 years ago, the workforce shortage has become more acute. A 2015 provider survey, which surveyed geneticists and genetic counselors, found that caseloads and patient wait times were increasing, but the capacity to see new patients was not.^13^ A 2015 consumer survey found that many patients reported difficulty finding a provider with expertise in their genetic condition, long wait times, and a lengthy diagnostic process.^14^ A recent study in California reported a medical genetics workforce shortage and significant geographic barriers in access to care.^15^ Additionally, a recently published systematic review indicated the shortage of genetics providers and lack of boundaries between scopes of practice for genetics and nongenetics providers and possible solutions such as alternative service delivery models, streamlining processes, and task automation.^16^ Finally, the US Government Accountability Office (GAO) published a report on 31 July 2020 that highlighted the recent modest increase in newly certified medical geneticists and the lack of clear data on demand for and the number of genetics professionals needed to provide genetic services.^17^

This article presents the findings of a 2019 US survey of medical geneticists conducted by the American College of Medical Genetics and Genomics (ACMG) through the National Coordinating Center (NCC) for the Regional Genetics Networks. The data in this paper focus on clinical geneticists in direct patient care. After characterizing the workforce shortage, we consider the means through which the workforce can be increased to meet today’s needs and those over the next ten years.

## MATERIALS AND METHODS

The “Current Practices in Medical Genetics (September 2019)” survey was hosted online via QuestionPro, an online survey tool. Two thousand four hundred five board-certified diplomates or board-eligible candidates received a link from the ABMGG via email. Three reminders were sent directly by ABMGG over the three-month survey period. ACMG, NCC, and the seven Regional Genetics Networks publicized the availability and importance of the survey to their members and stakeholders but did not share a separate link. Instead, they directed individuals to the ABMGG email. Only ABMGG had access to the list of individuals who received the survey and only ACMG had access to the survey tool and data. The emails sought to invite all medical geneticists, focusing specifically on clinical geneticists and their practice.

The survey (which can be accessed at https://nccrcg.org/wp-content/uploads/2019-NCC-Final-Survey.pdf) was fielded from 9 September 2019 through 9 December 2019 and was sent to ABMGG diplomates who were asked whether or not they were involved in clinical care. It contained 32 questions focused on two main areas: a respondent’s personal medical genetics training and clinical genetics practice information. This survey sought to mirror questions found in the prior medical geneticist workforce surveys.^9,13^

### Statistical analyses

Results were imported from QuestionPro into JMP (SAS) Pro (v.14.1.0) for tables, Statistical Package for Social Science (SPSS) and Excel for quantitative analysis, and R software for geocoding and map creation.

## RESULTS

### Demographic characteristics, education, and ABMGG certifications of clinical geneticists

A total of 984 medical geneticists responded to the survey (41% overall response rate) and this analysis focused largely on clinical genetics respondents (40% response rate). Clinical geneticists are defined here as any survey participant boarded in CGG through the ABMGG and currently practicing (actively seeing patients) (*n* = 491) in the United States. There were additional clinical geneticists (*n* = 69) who participated in the survey, but were not actively seeing patients and were subsequently excluded from these analyses. Clinical geneticists were largely female (59%) and White (79%). Respondents could identify with more than one race or ethnicity. Other clinical geneticists identified as Asian (11%), Hispanic (8%), or Black (1%). Average age for clinical geneticists was 51.4 years, with the oldest respondents indicating ages into the mid-80s.

A majority of responding clinical geneticists were certified by the ABMGG in CGG only (75.8%). The remainder were certified in additional areas, including MBG (12.4%), CBG (11.6%), CCyG (5.5%), and MGG (6.3%) (Table 1). Seventy-eight percent of clinical geneticists had an MD/DO and 21% held both MD/DO and PhD advanced degrees (Table 1).

**Table 1:** Clinical geneticists by American Board of Medical Genetics and Genomics (ABMGG) specialty/subspecialties and degree obtained.

Category	Clinical geneticists, *n* (%)
**ABMGG specialty**	***n*** = **491**
Clinical genetics and genomics (CGG) only	372 (75.8)
CGG and additional specialty/subspecialty^a^	
Medical biochemical genetics subspecialty	61 (12.4)
Clinical biochemical genetics	57 (11.6)
Clinical cytogenetics and genomics	27 (5.5)
Clinical molecular genetics and genomics	31 (6.3)
Laboratory genetics and genomics	0 (0.0)
Clinical biochemical/molecular (1990/1993 only)	11 (2.2)
PhD medical genetics	1 (0.2)
**Degree obtained**	***n*** = **490**
MD/DO	381 (77.8)
MD/DO and PhD	102 (20.8)
PhD	1 (0.2)
Other advanced degree	6 (1.2)

^a^Multiple additional specialties/subspecialties were indicated by respondents. Molecular genetic pathology subspecialty was removed for low participation.

### Years of practice and plans to retire

Length of practice for respondents reflects the age of the cohort; 36% of clinical geneticists have been practicing 21 years or more, while 21% of clinical geneticists indicated practicing less than five years (Table 2). Forty-four percent of clinical geneticists did not plan to reduce hours within the next ten years, while almost a quarter planned to retire in that same time period. Interestingly, there were several instances of retired clinical geneticists still seeing patients (Table 2).

**Table 2:** Years of practice and plans to retire.

Category	Clinical geneticists, *n* (%)
**Years of practice**	***n*** = **490**
Still in training	10 (2.0)
Less than 5 years	104 (21.2)
5–10 years	82 (16.7)
11–15 years	68 (13.9)
16–20 years	48 (9.8)
21 or more years	178 (36.3)
**Plan to reduce hours**	***n*** **= 486**
0–5 years	97 (20.0)
6–10 years	69 (14.2)
>10 years	216 (44.4)
Already retired	13 (2.7)
Don’t know	91 (18.7)
**Retirement**	***n*** **= 490**
0–5 years	57 (11.6)
6–10 years	59 (12.0)
>10 years	238 (48.6)
Already retired	4 (0.8)
Don’t know	132 (26.9)

### Practice characteristics

In the primary practice setting for clinical geneticists, 73% worked in academic medical centers, while others worked in private/group practice (11%), community hospitals (8.6%), or commercial laboratories (1%). A third of clinical geneticists engaged in telemedicine and approximately three-quarters indicated that they are required to be on call, with 92% reporting both telephone and in-person on-call duties (Table 3). On average, clinical geneticists spent 59 hours per week on call (telephone and in-person), with several respondents indicating being on call 24 hours a day, seven days a week (Table 3).

**Table 3:** Practice characteristics: setting, use of telemedicine, hours on call.

Category	Clinical geneticists, *n* (%)
**Primary practice setting**	***n*** **= 490**
Academic medical center	357 (72.9)
Private/group practice	54 (11.0)
Community hospital	42 (8.6)
Commercial laboratory	5 (1.0)
Other	32 (6.5)
**Practice telemedicine beyond main practice**	***n*** **= 472**
Yes	156 (33.1)
No	316 (66.9)
**Required to be on call as medical geneticist**	***n*** **= 472**
Yes	345 (73.1)
No	127 (26.9)
**Type of on call performed**	***n*** **= 349**
Telephone and in-person	321 (92.0)
Telephone only	28 (8.0)
**Number of average hours on call (telephone & in-person)**	***n*** **= 267**
Mean (±SD)	59.1 (±56.2)
Median (range)	40 (0-168)
**Number of average hours on call (telephone only)**	***n*** **= 27**
Mean (±SD)	51.3 (±61.9)
Median (range)	24 (0–168)

The majority (56.8%) of clinical geneticists spent more than 50% full-time equivalent (FTE) on medical genetics activities, including general genetics, cancer genetics, biochemical genetics, clinical laboratory testing, etc. (Table S1). Sixty percent of providers indicated working between 41 and 60 hours per work week, with an average of 50.2 hours (Table S1).

Most clinical geneticists (65%) spent a majority of their time providing care to patients (i.e., face-to-face and non-face-to-face care) (Figure S1). The average percent FTE spent on direct patient care among the clinical geneticist survey respondents was 58%. The majority of providers (86%) spent some of their time in pediatric genetics (Figure S2). Respondents also reported practicing adult medical, metabolic, and prenatal/reproductive genetics, though the amount of time spent in these areas varied greatly among providers. A minority of providers spent less than a quarter of their time providing patient care and, instead, devoted most of their time to research activities. Less than 20% of providers spent time in laboratory genetics. On average, clinical geneticists dedicated 10% of their time to both administrative and teaching/education activities.

### Practice capacity and trends

A large majority of clinical geneticists indicated that their practice is open to new patients (92%). This is a stark contrast to the 2015^13^ and 2003^9^ genetics workforce surveys that indicated only 28% and 32% of practices were accepting new patients, respectively (Table 4). While a majority of practices were still accepting patients, respondents indicated an increase in weekly patient visits and appointment wait times. In our study, during the portion of their time spent on patient care, clinical geneticists reported an average of 13 new patient visits per week, and ten follow-up patient visits per week. These numbers have steadily increased over time, with ten new and eight follow-up patient visits indicated per week in the 2015 workforce survey^13^ and six new and four follow-up patient visits in 2003^9^ (Table 4).

**Table 4:** Clinical geneticist practice capacity trends over time.

Category	(2019)	Maiese (2015)	Cooksey (2003)
**Accepting new patients**	***n*** = **442**	***n*** = **181**	***n*** = **376**
Open to new patients	92%	28%	32%
Practice nearly full	8	62	63
Not taking new patients	1	9	5
**Average number of patient visits/week**			
New patients	*n* = 419	*n* = 183	*n* = 315
Mean	12.5	10.2	6.0
Follow-up patients	*n* = 390	*n* = 183	*n* = 315
Mean	9.8	7.8	4.0
**Wait time, new nonemergency patient**	***n*** = **467**	***n*** = **210**	***n*** = **676**
1–2 days	2%	<10%	4%
3–6 days	3	6^a^	10^a^
1–3 weeks	15	11	38
1–3 months	36	32	36
More than 3 months	39	30	11
Not applicable	4	20	2
**Wait time, new emergency patient**	***n*** = **441**	-	-
Same day	31%	-	-
1–2 days	30	-	-
3–6 days	21	-	-
1–3 weeks	15	-	-
>1 month	3	-	-

^a^Wait times for new nonemergency patients was 3–5 days in Maiese (2015) survey and Cooksey (2003) survey and all days were specified work days. Dashes reflect no information in Maiese and Cooksey surveys to compared to survey results reported here.

Appointment wait times have increased compared to the previous studies. Thirty-nine percent of new nonemergency patients are waiting more than three months to be seen by a clinical geneticist, compared to 30% in 2015^13^ and 11% in 2003.^9^ While about a third of new emergency patients are seen the same day, 39% waited three days or longer to be seen (Table 4).

### Geographic distribution of clinical geneticists and job vacancies

**Fig. 1:**
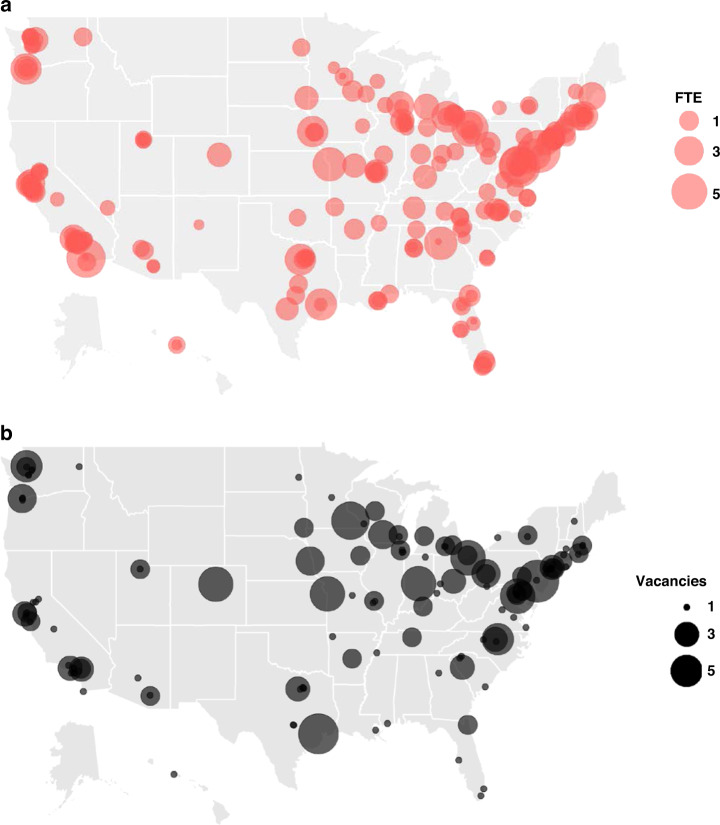
Geographic distribution of clinical geneticist respondents across the United States. Geocoded primary practice locations of geneticists who provide direct medical care by full-time equivalent (FTE) (**a**) and reported at least one job vacancy (**b**).

The reported FTE spent in medical genetics of survey respondents was used to map the geographic distribution of clinical geneticists by number of FTEs (Fig. 1a). Respondents were located in 45 states and were largely concentrated in major metropolitan areas or academic medical centers. This correlates with data provided by the ABMGG (M. Blitzer, unpublished data, April 2020) that demonstrated there are 14 states that had five or fewer currently certified clinical geneticists and one state that had none.

Respondents were asked to report clinical geneticist job vacancies at their practice. The length of these open vacancies ranged from 0–2 months to more than three years. The geographic locations of these vacancies followed a similar pattern as FTE distribution (Fig. 1b). Of note, some vacancies may be overrepresented because respondents who practice at the same location, and therefore have the same zip code, could have reported the same vacancy.

## DISCUSSION

This study reflects the current landscape of the US clinical genetics workforce, which has implications for access to care and the future of the specialty. In spite of rapid advances in genetic understanding, testing, and therapies, the clinical genetics workforce is not growing to address current patient and family needs. The future of genomic medicine will depend on a robust response to the current workforce shortage.

Our survey highlights several current trends in the medical genetics workforce in general. First, the average age of responding clinical geneticists is over 50 years, which reflects the current shortage of younger physicians who are seeking medical genetics residency training.^12^ Almost a quarter plan to retire within the next ten years. Second, the number of female clinical geneticists is increasing, up from 45% in 2003^9^ to 60% in our current survey, a trend that has also been observed in medical school admissions.^18^ Third, diversity continues to be an issue. Our survey indicates that fewer than 20% of respondents are non-White, which is considerably less than the 44% of non-White active US physicians in 2018.^18^ The lack of diversity among clinical geneticists, and the associated challenges of cultural competence, introduce potential barriers to care that can exacerbate existing racial and ethnic health-care disparities.

Almost three-quarters of clinical geneticists indicate practicing in academic medical centers, which provide care for the highest-acuity patients. Since the majority of providers work at large medical institutions, clinical genetics services are largely concentrated in urban areas, with 40% of providers located in only five states. Disparities in the geographic distribution of clinical geneticists limits rural and community-based access to genetics services. Geographic barriers to care can be partially addressed with the use of telemedicine, which has increased from 16% in 2015^13^ to 33% in our current survey. Telemedicine, however, is not sufficient to address the problem of inadequate numbers of geneticists to provide clinical service.

Although the average work week for clinical geneticists has decreased slightly from 2003^9^ (50 vs. 55 hours/week), the amount of time spent on call can range from a few hours a week to continuous service. Coupled with increasing patient visits per week (up from 6 new patient visits in 2003^9^ vs. 13 in our survey) and substantial wait times for both new emergency and nonemergency patients, the need for an expanded clinical geneticist workforce is clear. With increasing workloads and patient volumes, the large number of clinicians who indicated their practices were still taking new patients was surprising. This could speak to the culture of the medical system, which discourages turning away new patients and, instead, is driven by patient acquisition into primary care networks as a means to drive downstream revenue.^19^

Providers distribute their time across many clinical genetics areas, though a majority indicated spending some time in pediatric genetics. The small number of combined specialists (e.g., maternal–fetal medicine geneticists) were not asked to account for separate specialty activities. In addition to patient care, providers also split their time between research, administration, and teaching activities. This may leave other clinical genetics areas (i.e., adult medical, metabolic, and prenatal/reproductive) lacking the appropriate personnel to function at full capacity and requires geneticists to stretch their time and skills across many genetics-related activities.

While the number of newly certified ABMGG clinical geneticists in 2019 is the highest in the past two decades (M. Blitzer, ABMGG unpublished data, April 2020), this increase does not appear to be keeping pace with the demand for clinical genetics services. Many survey respondents identified open clinical geneticist positions at their centers, and several had been open for over one year. Since these openings generally track with FTEs reported by zip code, filling these clinical geneticist job vacancies may not substantially improve geographic disparity in access to care.

Studies that attempt to quantify current and future medical genetics workforce needs are few and limited. In 2013, the Royal College of Physicians of the United Kingdom reaffirmed that their health-care system required 0.75 clinical geneticist FTE per 250,000 individuals (3 per 1 million).^20^ The 2020 GAO report indicated that there was 1 medical geneticist per 250,000 in the US population (4 per 1 million), although that number reflects clinical geneticists, not all medical geneticists (the report used the terms medical and clinical geneticists interchangeably).^17^ Current estimates from this survey and ABMGG place that number between 1.9 and 2.2 clinical geneticist FTE per 1 million. Results of this survey found that clinical geneticists, on average, spend 58% of their time in direct patient care suggesting that the United States has 2.2 clinical geneticist FTE per million in the US population. ABMGG data suggests the United States currently has 1.9 clinical geneticist FTE per 1 million people (M. Blitzer, unpublished data, April 2020). Furthermore, because the ABMGG denominator used to ascertain this rate represents diplomates who are currently certified without regard to their status in the workforce, this could be an overestimate of those currently in the workforce. The survey results presented here could be an overestimate since not all clinical geneticists responded to the survey. This contrast is made more dramatic when considering the practice differences that exist between the UK and United States. Patients referred to a clinical geneticist in the UK are typically evaluated for a diagnosis and then referred back to primary care for ongoing follow-up. Clinical geneticists in the United States, on the other hand, typically follow patients with a genetics diagnosis indefinitely, often for decades. For example, patients with inborn errors of metabolism (IEM) in the UK are evaluated and treated by a different specialty entirely, metabolic medicine, for which no recommended number exists. In the United States, IEM patients are evaluated and followed by specially trained clinical geneticists. While there exists no established number of specialists needed for all clinical genetics services in the United States, all indications suggest that there are inadequate numbers of clinical geneticists to provide for current needs. Future studies must assess these needs, and address shortages, if we hope to fulfill the promises of personalized medicine.

### Looking ahead

To address the current shortfall and expected capacity needs of genomic medicine, a substantial increase in clinical genetics trainees will be necessary. However, there have been persistent deficiencies in filling training slots, which was highlighted in the first Banbury Conference more than 15 years ago.^10^ While there has been an increase of total residents in clinical genetics residency programs (when looking at residents in categorical and combined programs combined), approximately 33% of the approved and funded clinical genetics residency positions remain unfilled (M. Blitzer, unpublished data, December 2020), impeding growth of the clinical genetics workforce.

Increasing the number of clinical geneticists alone will not fully solve practice capacity issues in the field, as clinicians work within a network of health-care professionals to provide comprehensive patient care. The medical genetics service delivery team includes other important provider groups not certified by ABMGG, such as genetic counselors, metabolic dietitians, physician assistants, nurses, researchers, and nongenetics trained physicians and providers. Working as an integrated medical team will require increased collaborative efforts across disciplines, such as the National Human Genome Research Institute’s Inter-Society Coordinating Committee for Practitioner Education in Genomics (ISCC-PEG).^21^ ISCC-PEG aims to facilitate interactions among various provider groups to improve their genomic literacy and enhance quality of care for a growing number of genetics patients.^22^

Genetic diseases affect all populations, but there is little diversity among the ranks of clinical geneticists. As the genetics workforce evolves over time, there is a prime opportunity to recruit trainees of various backgrounds to match growing diversity in the United States and provide unique insights into patient interaction and care.^23^ Practices should consider organization-wide cultural competency training and development, so they can better incorporate patient-centered care into their practice. Genetics organizations may benefit from forming diversity committees to examine whether medical ethical principles and procedures are applied equitably across all races and cultures at their institution.

The inequitable geographic distribution of clinical geneticists must be addressed to make clinical genetics health care more accessible. Telemedicine is helping to complement in-person clinical genetics services, and the recent expansion of telehealth during the COVID-19 pandemic has integrated telemedicine into routine clinical practice. Appropriate reimbursement for telehealth and established systems to manage these services are critical during these unprecedented times and for the future of the field. Genetics providers must also be willing to permanently adopt telemedicine into their routine practice.^24^

### Study limitations

This study has several limitations. First, the survey’s response rate reflects the challenge of achieving robust clinician participation. This may, in part, reflect the clinician’s lack of time to respond owing to workload and survey fatigue. The response rate could also lead to nonresponse bias and may limit the generalizability of the findings. Second, more descriptive techniques were used in this analysis versus more complex statistical methods to allow readers to easily probe for questions of interest. Finally, the survey presents some variability in item response, as respondents were not required to answer every question. Additionally, comparisons were made between past medical genetics workforce surveys, and while some questions were equivalent, others were presented differently between surveys and not directly comparable. Overall, however, there was consensus among the NCC working group that these findings accurately reflect the current perceptions of the medical genetics workforce.

### Conclusions

These survey results reinforce the continuing clinical genetics workforce shortages and capacity limitations. Potential solutions, such as increasing the recruitment of clinical genetics trainees into the field, improving workforce diversity, and enhancing collaborative practice are a start, but will require a concerted effort and innovations across many stakeholders to fully realize. Salary enhancement and increased funding support for trainees will be integral in achieving this goal. If the workforce is successfully enhanced, genetics patients will benefit more from cutting edge research and therapies, in addition to a more collaborative approach to medical genetics care.

## Supplementary information

Supplementary Information

## Data Availability

Summary data is available by contacting ACMG and NCC staff: ncc@nccrcg.org.
