# Supplementary material for: The 2019 US medical genetics workforce: a focus on clinical genetics
Source: Genet Med. 2021 May 3;23(8):1458–64. doi: 10.1038/s41436-021-01162-5 (PMC8091643; doi:10.1038/s41436-021-01162-5)
Supplement: Supplementary file 1 — Supplementary Information [file 41436_2021_1162_MOESM1_ESM.pdf]

Table S1. Work Hours Spent

Category	Clinical Geneticists, n (%)
**% FTE on medical genetics**	**n=491**
<50%	204 (41.5)
>50%	279 (56.8)
Not Applicable	8 (1.6)
**No. of hours in avg work week**	**n=460**
Mean (±SD)	50.2 (±15.0)
Median (Range)	50 (0-120)
0-20	24 (5.2)
21-40	99 (21.5)
41-60	275 (59.8)
>61	61 (13.3)
Don't Know/Not Applicable	1 (0.2)

FTE, full time equivalent, SD, standard deviation

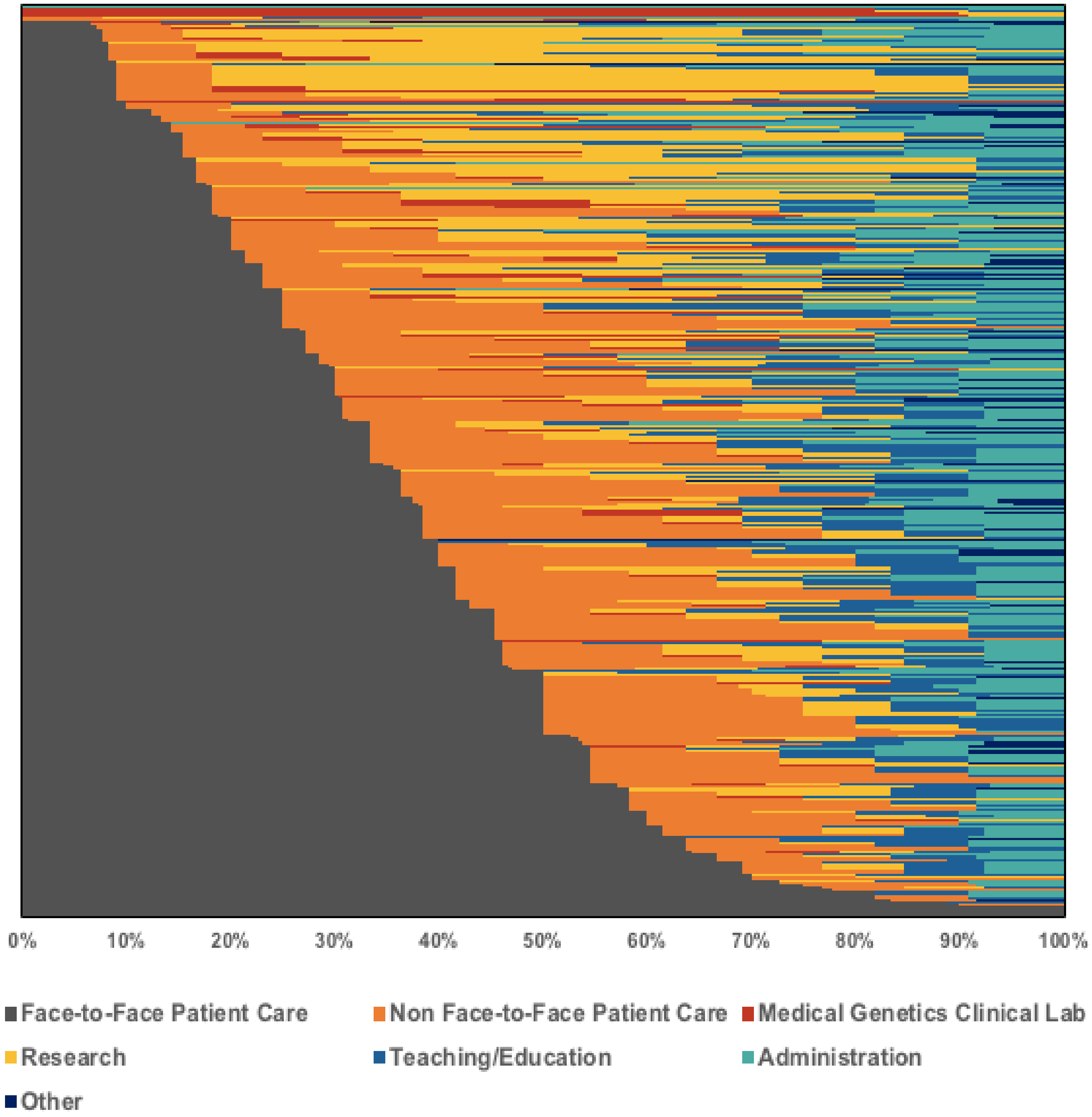

Figure S1. Distribution of percent time spent in genetics-related activities (n=432). Percent time was normalized to 100%

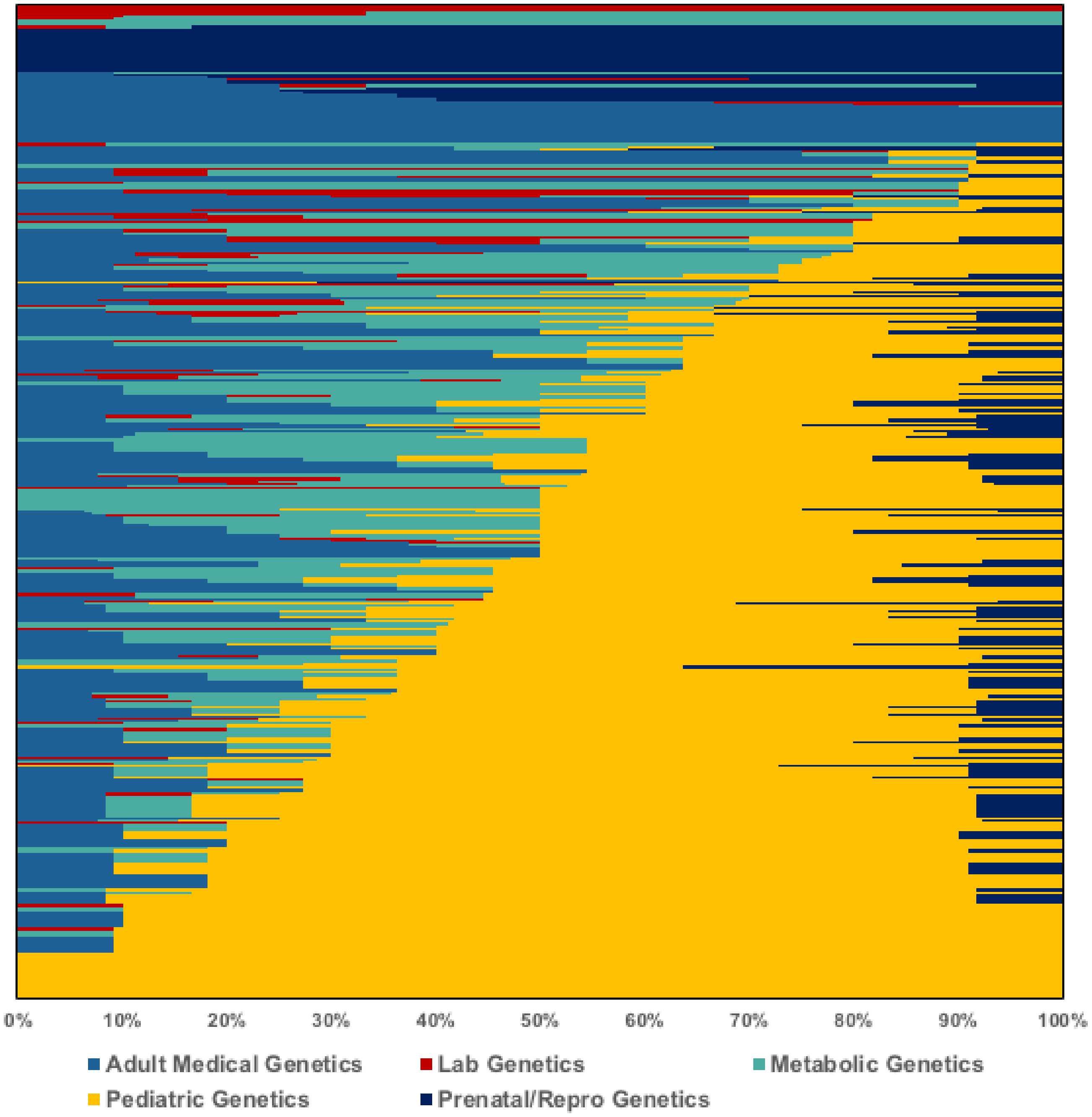

Figure S2. Distribution of percent time spent in clinical genetics areas (n=439). Percent time was normalized to 100%

NCC Genetics Workforce Working Group Roster

Hans Andersson, MD
Tulane University

Judith Benkendorf, MS,CGC
American College of Medical Genetics and Genomics

Miriam Blitzer, PhD, MS
American Board of Medical Genetics and Genomics

Joann Bodurtha, MD, MPH
Johns Hopkins University

Tina Cowan, PhD
Stanford University

Andrea Durst, MS, DrPH, LCGC
University of Pittsburgh

Mat Edick, PhD
Michigan Public Health Institute

Jennifer Hoskovec, MS, CGC
University of Texas Health Science Center at Houston

Alisha Keehn, MPA
American College of Medical Genetics and Genomics/National Coordinating Center for the Regional Genetics Networks and Health Resources and Services Administration/Maternal and Child Health Bureau (as of 11/08/20)

Mark Korson, MD
VMP Genetics

Megan Lyon, MPH
American College of Medical Genetics and Genomics/National Coordinating Center for the Regional Genetics Networks

Debbie Maeise, MPA
American College of Medical Genetics and Genomics/ National Coordinating Center for the Regional Genetics Networks

Cecily Marroquin, MA
American Board of Medical Genetics and Genomics

Shawn McCandless, MD
Children's Hospital of Colorado

Michelle McClure, PhD
American College of Medical Genetics and Genomics

Honey V Reddi, PhD
Medical College of Wisconsin

John Richardson
National Society of Genetic Counselors

Rani Singh, PhD, RD, LD
Emory University

Joseph Shieh, MD, PhD
University of California San Francisco

Matthew Taylor, MD, PhD
University of Colorado Denver

Michael Watson, PhD, MS
American College of Medical Genetics and Genomics

Lori Williamson Dean, MS, CGC
University of Arkansas for Medical Sciences
